# Supplementary material for: Association between serum ferritin and mortality in patients with severe fever with thrombocytopenia syndrome: A retrospective cohort study
Source: PLoS Negl Trop Dis. 2025 May 22;19(5):e0013104. doi: 10.1371/journal.pntd.0013104 (PMC12129351; doi:10.1371/journal.pntd.0013104)
Supplement: S1 Table — (DOCX) [file pntd.0013104.s001.docx]

| **Variable** | **HR** | **95%CI** | ***P*** |
| --- | --- | --- | --- |
| **Demographic feature** |  |  |  |
| Gender, Male | 1.498 | 0.961-2.335 | 0.075 |
| Age | 1.071 | 1.043-1.110 | <0.001 |
| Farmer | 0.746 | 0.418-1.331 | 0.321 |
| **Symptoms** |  |  |  |
| Diarrhea | 1.385 | 0.888-2.160 | 0.151 |
| Pancreatitis | 3.580 | 2.065-6.205 | <0.001 |
| Neurologic symptoms | 97.032 | 30.553-308.153 | <0.001 |
| Gastrointestinal bleeding | 6.233 | 3.970-9.788 | <0.001 |
| Pulmonary fungal infection | 6.776 | 4.244-10.819 | <0.001 |
| Bacteremia | 2.783 | 1.501-5.160 | 0.001 |
| **Comorbidity** |  |  |  |
| Hypertension | 2.173 | 1.393-3.390 | <0.001 |
| Diabetes | 1.099 | 0.529-2.284 | 0.800 |
| Stroke | 2.510 | 1.408-4.476 | 0.002 |
| **Vital signs** |  |  |  |
| Temperature | 1.219 | 0.966-1.537 | 0.095 |
| Respiratory rate | 1.124 | 1.048-1.206 | 0.001 |
| Heart rate | 1.028 | 1.016-1.041 | <0.001 |
| Systolic BP | 1.016 | 1.004-1.028 | 0.008 |
| Diastolic BP | 1.019 | 0.998-1.041 | 0.078 |
| **Laboratory tests** |  |  |  |
| SFTSV RNA | 2.393 | 2.003-2.859 | <0.001 |
| White blood cell | 0.987 | 0.911-1.068 | 0.739 |
| Hemoglobin | 1.003 | 0.991-1.015 | 0.618 |
| Platelet | 0.981 | 0.971-0.992 | <0.001 |
| Glutamic-pyruvic transaminase | 1.001 | 1.001-1.001 | <0.001 |
| Total bilirubin | 1.036 | 0.995-1.079 | 0.087 |
| Creatinine | 1.009 | 1.007-1.011 | <0.001 |
| Urea nitrogen | 1.146 | 1.114-1.178 | <0.001 |
| Lactic dehydrogenase | 1.001 | 1.000-1.001 | <0.001 |
| Creatine kinase | 1.000 | 1.000-1.000 | 0.004 |
| Creatine kinase MB | 1.001 | 1.000-1.001 | <0.001 |
| Plasma prothrombin time | 1.266 | 1.150-1.393 | <0.001 |
| D-D dimer | 1.037 | 1.025-1.049 | <0.001 |
| Thrombin time | 1.018 | 1.012-1.023 | <0.001 |
| Fibrinogen | 0.497 | 0.323-0.764 | 0.001 |
| C-reactive protein | 1.009 | 1.003-1.015 | 0.005 |
| Procalcitonin | 1.127 | 1.074-1.182 | <0.001 |
| Serum ferritin | 5.950 | 4.059-8.720 | <0.001 |
