# Supplementary material for: Association between serum ferritin and mortality in patients with severe fever with thrombocytopenia syndrome: A retrospective cohort study
Source: PLoS Negl Trop Dis. 2025 May 22;19(5):e0013104. doi: 10.1371/journal.pntd.0013104 (PMC12129351; doi:10.1371/journal.pntd.0013104)
Supplement: S2 Table — (DOCX) [file pntd.0013104.s002.docx]

| **Variable** | **HR** | **95%CI** | ***P*** |
| --- | --- | --- | --- |
| Gender, Male | 0.966 | 0.612-1.527 | 0.884 |
| Age | 1.065 | 1.036-1.095 | <0.001 |
| Farmer | 0.654 | 0.362-1.183 | 0.160 |
| Serum ferritin | 5.968 | 3.969-8.975 | <0.001 |

Model I: Adjusted for age, sex, and profession.
