# Supplementary material for: Association between serum ferritin and mortality in patients with severe fever with thrombocytopenia syndrome: A retrospective cohort study
Source: PLoS Negl Trop Dis. 2025 May 22;19(5):e0013104. doi: 10.1371/journal.pntd.0013104 (PMC12129351; doi:10.1371/journal.pntd.0013104)
Supplement: S3 Table — (DOCX) [file pntd.0013104.s003.docx]

| **Variable** | **HR** | **95%CI** | ***P*** |
| --- | --- | --- | --- |
| Gender, Male | 0.935 | 0.589-1.486 | 0.778 |
| Age | 1.061 | 1.031-1.092 | <0.001 |
| Farmer | 0.772 | 0.389-1.340 | 0.302 |
| Hypertension | 1.296 | 0.802-2.094 | 0.290 |
| Diabetes | 0.969 | 0.461-2.036 | 0.934 |
| Stroke | 1.304 | 0.690-2.462 | 0.413 |
| Serum ferritin | 5.714 | 3.780-8.639 | <0.001 |

Model II: Adjusted for age, sex, profession, and comorbidity.
