# Supplementary material for: Association between serum ferritin and mortality in patients with severe fever with thrombocytopenia syndrome: A retrospective cohort study
Source: PLoS Negl Trop Dis. 2025 May 22;19(5):e0013104. doi: 10.1371/journal.pntd.0013104 (PMC12129351; doi:10.1371/journal.pntd.0013104)
Supplement: S4 Table — (DOCX) [file pntd.0013104.s004.docx]

| **Variable** | **HR** | **95% CI** | ***P*** |
| --- | --- | --- | --- |
| **Demographic feature** |  |  |  |
| Gender, Male | 0.372 | 0.195-0.712 | 0.003 |
| Age | 1.054 | 1.006-1.104 | 0.026 |
| Farmer | 0.659 | 0.297-1.465 | 0.307 |
| **Symptoms** |  |  |  |
| Diarrhea | 0.622 | 0.352-1.101 | 0.103 |
| Pancreatitis | 0.426 | 0.209-0.871 | 0.019 |
| Neurologic.symptoms | 60.281 | 16.431-221.160 | <0.001 |
| Gastrointestinal.bleeding | 0.922 | 0.492-1.730 | 0.801 |
| Pulmonary.fungal.infection | 1.957 | 1.040-3.682 | 0.037 |
| Bacteremia | 1.189 | 0.520-2.720 | 0.682 |
| **Comorbidity** |  |  |  |
| Hypertension | 0.792 | 0.404-1.552 | 0.496 |
| Diabetes | 0.881 | 0.370-2.099 | 0.776 |
| Stroke | 0.687 | 0.304-1.551 | 0.366 |
| **Vital signs** |  |  |  |
| Temperature | 1.393 | 1.001-1.938 | 0.050 |
| Respiratory rate | 0.902 | 0.782-1.042 | 0.160 |
| Heart rate | 1.015 | 0.995-1.035 | 0.143 |
| Systolic BP | 0.999 | 0.978-1.021 | 0.959 |
| Diastolic BP | 1.014 | 0.976-1.054 | 0.468 |
| **Laboratory tests** |  |  |  |
| SFTSV RNA | 1.332 | 1.036-1.712 | 0.025 |
| White blood cell | 1.034 | 0.890-1.201 | 0.664 |
| Hemoglobin | 1.006 | 0.988-1.024 | 0.522 |
| Platelet | 0.988 | 0.972-1.004 | 0.136 |
| Glutamic-pyruvic transaminase | 1.001 | 1.000-1.002 | 0.083 |
| Total bilirubin | 1.031 | 0.953-1.115 | 0.448 |
| Creatinine | 1.010 | 1.003-1.016 | 0.006 |
| Urea nitrogen | 0.996 | 0.920-1.078 | 0.918 |
| Lactic dehydrogenase | 0.999 | 0.998-1.00 | 0.002 |
| Creatine kinase | 1.000 | 1.000-1.000 | 0.388 |
| Creatine kinase MB | 1.001 | 0.999-1.002 | 0.201 |
| Plasma prothrombin time | 1.192 | 1.004-1.415 | 0.045 |
| D-D dimer | 0.982 | 0.956-1.010 | 0.208 |
| Thrombin time | 0.999 | 0.987-1.010 | 0.803 |
| Fibrinogen | 0.778 | 0.454-1.332 | 0.360 |
| C-reactive protein | 1.007 | 0.995-1.020 | 0.227 |
| Procalcitonin | 0.907 | 0.805-1.023 | 0.111 |
| Serum ferritin | 5.982 | 2.752-13.004 | <0.001 |

Model III: Adjusted for all covariates.
