# Supplementary figures and images for: Association between serum ferritin and mortality in patients with severe fever with thrombocytopenia syndrome: A retrospective cohort study
Source: PLoS Negl Trop Dis. 2025 May 22;19(5):e0013104. doi: 10.1371/journal.pntd.0013104 (PMC12129351; doi:10.1371/journal.pntd.0013104)

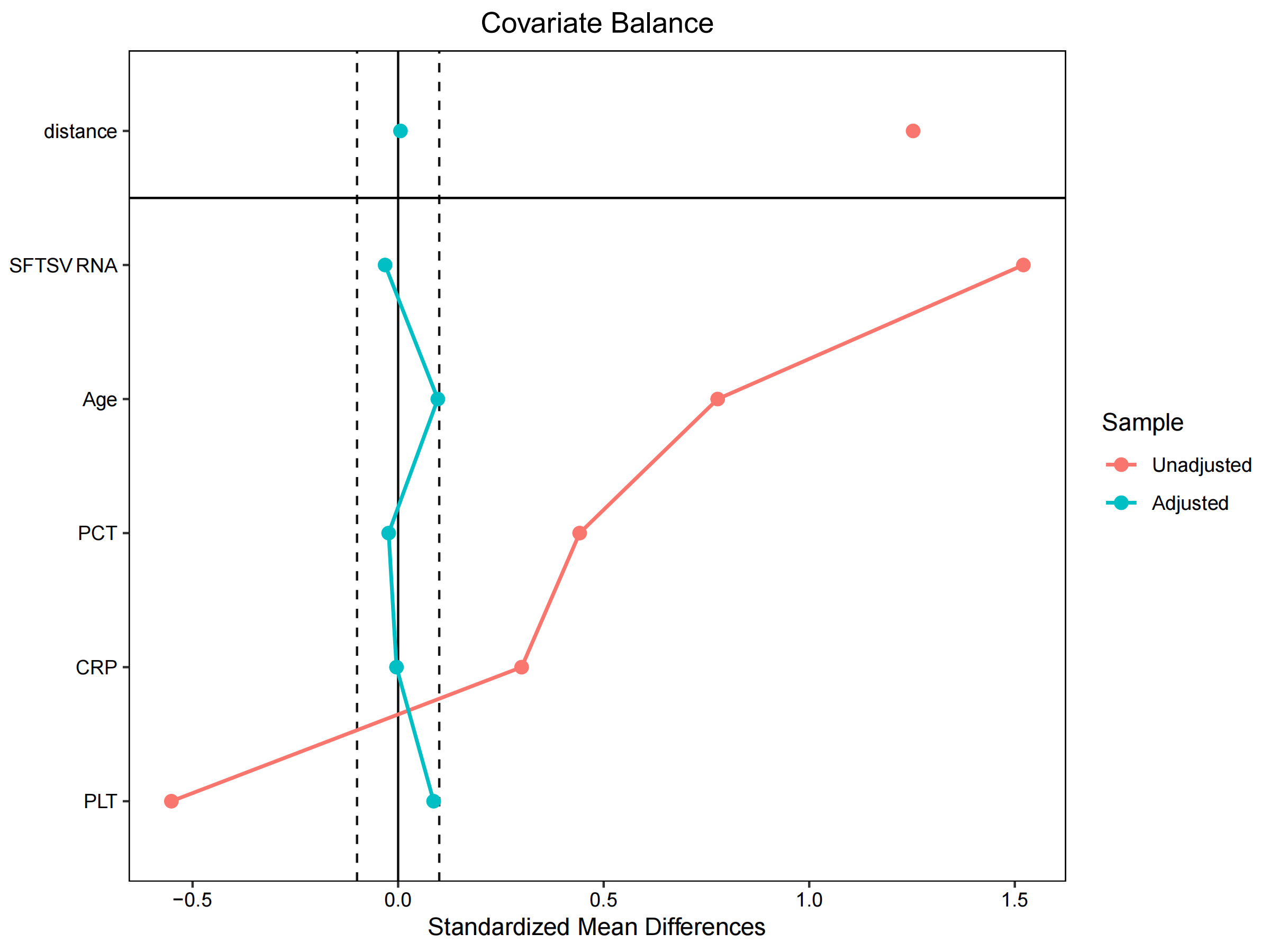

Supplement: S1 Fig — (TIF) [file pntd.0013104.s005.tif]
