# Supplementary material for: Association between serum ferritin and mortality in patients with severe fever with thrombocytopenia syndrome: A retrospective cohort study
Source: PLoS Negl Trop Dis. 2025 May 22;19(5):e0013104. doi: 10.1371/journal.pntd.0013104 (PMC12129351; doi:10.1371/journal.pntd.0013104)
Supplement: Attachment — Submitted filename: Response letter to the Editors and Reviewers.docx [file pntd.0013104.s009.docx]

**Dear editor and reviewers,**

**On behalf of my co-authors, we greatly appreciate the careful review and comments from you and the three reviewers. We believe that by implementing the suggested changes, we now have a stronger manuscript entitled “*Association between serum ferritin and mortality in patients with severe fever with thrombocytopenia syndrome：a retrospective cohort study*” (Manuscript Number: PNTD-D-24-01734) for submission to PLOS Neglected Tropical Diseases. We look forward to your positive response to the revised work submitted here.**

**We present here point-to-point responses for each of the comments in the attached document and have revised our manuscript accordingly. We do not change our statistics or results. And we hope the revised manuscript could be acceptable for you. Revised sections are identified with red text in the paper.**

**There are no conflicts of interest regarding this work. All authors have read the revised manuscript and approved its submission to the Journal of Critical Care. Please do not hesitate to contact us if we can be of any further assistance.**

**Thank you and best regards.**

**Your Sincerely,**

**Min Yang, Prof. PhD, E-mail: yangmin@ahmu.edu.cn.**

**The Second Department of Critical Care Medicine, the Second Affiliated Hospital of Anhui Medical University, No.678 Furong Road, Hefei, Anhui, China**

***Below I will respond to the comments of editors and reviewers one on one.***

***Journal Requirements:***

1) ***Tables should not be uploaded as individual files. Please remove these files and include the Tables in your manuscript file as editable, cell-based objects. For more information about how to format tables, see our guidelines:<https://journals.plos.org/plosntds/s/tables>***

Reply: Thank you for your careful review. We have modified according to the manuscript requirements and put the editable cell-based table in the manuscript. Thank you again for your reminding.

2) ***We have noticed that you have uploaded Supporting Information files, but you have not included a list of legends. Please add a full list of legends for your Supporting Information files after the references list.***

Reply: Thank you for your review. We have placed the legend list behind the references as you reminded us.

3) ***In the online submission form, you indicated that "The dataset used in this study is available from the corresponding author upon reasonable request". All PLOS journals now require all data underlying the findings described in their manuscript to be freely available to other researchers, either
   - In a public repository
   - Within the manuscript itself
   - Uploaded as supplementary information.
   This policy applies to all data except where public deposition would breach compliance with the protocol approved by your research ethics board. If your data cannot be made publicly available for ethical or legal reasons (e.g., public availability would compromise patient privacy), please explain your reasons by return email and your exemption request will be escalated to the editor for approval. Your exemption request will be handled independently and will not hold up the peer review process, but will need to be resolved should your manuscript be accepted for publication. One of the Editorial team will then be in touch if there are any issues.***

Reply: Thank you very much for your reminding. We have uploaded all the data described in the manuscript to the system as supplementary information, and in the manuscript we have revised the section "Availability of data and material declaration" and noted that "All relevant data are within the manuscript and it’s Supporting Information files". Thank you again for your review.

***4) Please amend your detailed Financial Disclosure statement. This is published with the article. It must therefore be completed in full sentences and contain the exact wording you wish to be published. Please ensure that the funders and grant numbers match between the Financial Disclosure field and the Funding Information tab in your submission form. Note that the funders must be provided in the same order in both places as well.
- State the initials, alongside each funding source, of each author to receive each grant. For example: "This work was supported by the National Institutes of Health (####### to AM; ###### to CJ) and the National Science Foundation (###### to AM)."
- State what role the funders took in the study. If the funders had no role in your study, please state: "The funders had no role in study design, data collection and analysis, decision to publish, or preparation of the manuscript.".
If you did not receive any funding for this study, please simply state: u201cThe authors received no specific funding for this work.u201d***

Reply: Thank you for your careful review. We modified the source of funding in the manuscript and identified it in red font. Thanks again for reminding me.

***Reviewers' Comments:***

***Methods****:*

***Reviewer #1: T***

Reply: Thank you for your careful review.

***Reviewer #2: Wenyan Xiao et al retrospective analysis using data from patients diagnosed with SFTS at the Second Affiliated Hospital of Anhui Medical University,and found the association between serum ferritin levels and in-hospital mortality in patients with SFTS, a study with a relatively large sample, also had some important findings.***

Reply: We appreciate your careful review and professional comments on our article.

***Reviewer #3: (No Response)***

Reply: Thank you for your careful review.

***Results****:*

***Reviewer #1: (No Response)***

Reply: Thank you for your careful review.

***Reviewer #2: 1) Single-center, retrospective study.
2) The changes of serum ferritin levels were not dynamically displayed and analyzed.***

Reply: We greatly appreciate your professional comments on our manuscripts. As you are concerned, these two issues are indeed the limitations of this study. Although this study was a single-center study with a relatively large sample size, due to its retrospective nature, it was not able to conduct a dynamic analysis of serum ferritin changes. In order to further confirm the evaluation value of serum ferritin in SFTS, our team is organizing a multicenter prospective study, and we believe that there will be sufficient evidence to prove this conclusion in the future. In addition, we have revised the limitations in the manuscript and will "require prospective randomized controlled trials to confirm the effect of serum ferritin levels on prognosis in patients with SFTS" Modified to read "Future multicenter prospective trials are needed to confirm the effect of serum ferritin levels on prognosis in patients with SFTS" (indicated in red font). Thank you again for your review.

***Reviewer #3: After PSM, SMD should be reported.***

Reply: Thank you for your careful review. We have made changes to the manuscript by adding SMD values (identified in blue) in Table 3. Thank you very much for your advice.

***Conclusions****:*

***Reviewer #1: (No Response)***

Reply: Thank you for your careful review.

***Reviewer #2: 3) The stage changes of acute viral infection and the course of the disease significantly affect the results of laboratory tests, that is, in the critical stage of SFTS infection, especially when accompanied by multiple organ damage such as pancreatitis, encephalitis, etc., the inflammatory markers and serum ferritin levels of patients will be significantly increased.***

Reply: We greatly appreciate your professional comments on our manuscripts. Our clinical findings are quite consistent with your opinion, that is, the different stages and course of STFS infection, especially when patients are complicated with multiple organ injuries such as pancreatitis and encephalitis, will indeed lead to a significant increase in inflammatory factors and serum ferritin, and we also find that such patients tend to have poor prognosis and high mortality. In this study, all the serum ferritin we included were collected within 24 hours of admission, and early detection of SF can predict the prognosis of patients, which precisely indicates that serum ferritin has a good predictive value for the prognosis of patients, which is not only conducive to early assessment of the disease by clinicians, but also conducive to early intervention of the disease. Thank you again for your review.

***4) Therefore, the level of serum ferritin is uncertain in the course of disease, but it will obviously increase in the case of multiple organ inflammation. At this time, the predictive role of serum ferritin is not very important.***

Reply: Thank you very much for your careful review. As you said, serum ferritin levels are uncertain over the course of the disease. The early increase in the level of ferritin in patients, regardless of whether it is induced by viruses or caused by multiple organ inflammation, if the early detection of elevated ferritin (within 24 hours of admission) can not only help clinicians predict the prognosis of patients, but also be very beneficial to clinicians' evaluation and intervention of the disease, thus highlighting the predictive value of serum ferritin. Therefore, we believe that the value of serum ferritin is very important for the prediction of SFTS patients. Thanks again for your comments.

***Reviewer #3: Further explanation is needed regarding why serum ferritin is associated with increased mortality. Is it due to sepsis (DOI: 10.3389/fnut.2021.747547) or cytokine storm (DOI: 10.1038/s41467-022-34560-7; 10.1038/s41584-022-00899-w)?***

Reply: Thank you very much for your valuable advice. Based on the literature study, we believe that there are two main reasons for the correlation between serum ferritin and increased mortality. On the one hand, serum ferritin may directly participate in the formation of cytokine storm, and then induce systemic inflammatory response, leading to multiple organ dysfunction; On the other hand, virus-related sepsis may be induced by SFTSV, aggravating organ function damage and thus increasing the mortality of patients. We have added the above discussion to the discussion section of the manuscript. Thanks again for your advice.

***Editorial and Data Presentation Modifications?***

***Reviewer #1: The paper reported an association between serum ferritin and mortality in patients with severe fever with thrombocytopenia syndrome (SFTS). It was found that serum ferritin levels are linearly associated with the mortality risk in SFTS patients. Specifically, when serum ferritin levels exceed 10,000 ng/ml, the mortality rate significantly increases. Thus, serum ferritin levels may serve as a valuable prognostic biomarker for assessing the mortality risk in SFTS patients. The study is well - designed, and its results are highly useful for predicting the prognosis of SFTS.
1. Results: Regarding the results section, particularly the "receiver operating characteristic (ROC) curve of serum ferritin for predicting in - hospital mortality in patients with SFTS", the authors are recommended to offer more detailed information. This could include aspects such as the area under the curve (AUC) value, the optimal cut - off point determined from the ROC analysis, and the sensitivity and specificity values at different thresholds. Such additional details would enhance the comprehensiveness and interpretability of the results.***

Reply: Thank you very much for your careful review and valuable comments. We provide data on the sensitivity and specificity of the AUC and different cutoff values in the supplementary material. These data help to improve the comprehensiveness and interpretability of the results. Thank you again for your valuable comments.

***2. Serum ferritin is generally regarded as having both pro - inflammatory and anti - inflammatory properties. Its function is intricate and context - dependent. As a result, it cannot be simply classified as a strictly pro - inflammatory or anti - inflammatory marker. In different physiological and pathological conditions, serum ferritin can exhibit diverse effects on the inflammatory response. For example, in certain acute - phase responses, it may act as a pro - inflammatory factor by being upregulated in response to inflammatory cytokines. However, in maintaining iron homeostasis, it can play an anti - inflammatory role by regulating the availability of iron, which is crucial for immune cell function and the prevention of oxidative stress - related inflammation.***

Reply: We appreciate your professional comments. We very much agree with you that serum ferritin is generally considered to have both pro-inflammatory and anti-inflammatory properties. Its function is complex and context-dependent. Therefore, it cannot simply be classified as a strictly pro-inflammatory or anti-inflammatory marker. Under different pathophysiological conditions, the effect of serum ferritin on systemic inflammatory response is indeed different. Even studies like Jia et al. believe that ferritin is directly involved in the formation of cytokine storms, inducing systemic inflammatory responses in the body, and has a powerful pro-inflammatory effect. At the same time, ferritin plays an anti-inflammatory role by regulating the availability of iron, which is essential for immune cell function and the prevention of oxidative stress-related inflammation. Therefore, the pathogenic role of ferritin in SFTS remains unclear and needs to be further studied. In the future, our team will carry out multi-omics studies on SFTS in order to elucidate the related pathophysiological mechanisms.

***3. Throughout the text, some minor adjustments have been made to enhance the fluency and clarity of the English.***

Reply: Thank you very much for your careful review. We made some adjustments to the entire text, and invited native English experts to study the revision and polishing. Thank you again for your review.

***Reviewer #2: (No Response)***

Reply: Thank you very much for your careful review.

***Reviewer #3: (No Response)***

Reply: Thank you very much for your careful review.

***Summary and General Comments****:*

***Reviewer #1: (No Response)***

Reply: Thank you very much for your careful review.

***Reviewer #2: (No Response)***

Reply: Thank you very much for your careful review.

***Reviewer #3: (No Response)***

Reply: Thank you very much for your careful review.
